# The study of waiting time to first pregnancy in the south of Iran: A parametric frailty model approach

**Published:** 2017-01

**Authors:** Najaf Zare, Bijan Nouri, Fariba Moradi, Maryam Parvareh

**Affiliations:** 1 *Department of Biostatistics, Infertility Research Center* *,* * Shiraz University of Medical Sciences, Shiraz, Iran.*; 2 *Social Determinants of Health Research Center, Kurdistan University of Medical Sciences, Sanandaj, Iran.*; 3 *Family Health Unit* *,* * The Office of Vice Chancellor for Health Affairs* *,* * Shiraz University of Medical Sciences* *,* * Shiraz, Iran.*

**Keywords:** Survival models, Frailty models, Fertility, Time to pregnancy

## Abstract

**Background:**

Time to first pregnancy (TTFP) has never been studied in an Iranian setting. Lifestyle, occupational and environmental factors have been suggested to affect the female reproduction.

**Objective:**

This study was conducted to measure TTFP in the south of Iran and survey the effects of several similar factors on TTFP by frailty models.

**Materials and Methods:**

The data on TTFP were available for 882 women who were randomly selected from the rural population (the south of Iran). Only the first and the planned pregnancies of every woman were included. The data were collected retrospectively by using self-administered questionnaires. Frailty and shared frailty models were used to determine which factors had the highest impact on TTFP.

**Results:**

The median TTFP was 6.4 months and several factors were surveyed. However, only the age of marriage, height, maternal education and regularity of menstruation prior to conception were selected in the multivariable models.

**Conclusion:**

Among the several factors which were included in the study, the result of frailty model showed that the height, age of marriage and regular menstruation seemed more notable predictors of TTFP.

## Introduction

Hfactors of human health and the survival of the species. It is defined as the capacity to produce offspring. Fertility, meanwhile, is defined as a woman’s biological ability to reproduce based on the monthly chance of conception. Time to pregnancy (TTP), the number of menstrual cycles take a couple engaging in normal unprotected sexual intercourse to conceive, is potentially a very informative measure of human fertility and an estimate for fecundity. 

However, TTP is an important problem for couples and contributes substantially to the population growth. To determine TTP, the starting dates of unprotected cohabitation and dates of conception are required. Time to first pregnancy (TTFP) is a special case of TTP and measures time to the first conception. TTFP data can be used to study the effects of environmental and occupational exposures on human fertility. The TTFP distribution can be studied using either a prospective or retrospective design. Environmental exposures, socio-demographic factors, behavioral and biological characteristics of couples may affect human reproduction via a range of diverse mechanisms leading to a common observable effect: whether a longer or shorter time is needed, on average, for affected couples to achieve conception.

Information on fertility health outcomes, involving TTFP and pregnancy outcomes can readily be collected by means of a questionnaire (1, 2). Some of the factors investigated for potential influence on fertility are reported as: lifestyle-related factors, such as alcohol consumption and smoking habits, biological and sexual behavior, age, environmental exposures, cultural background, geographical location and knowledge (2-6). The effects of certain covariates (in particular, the age of a woman) are related to both biological and behavioral aspects of fecundity (7). 

The relationship between height and reproductive success (RS) is not same for both males and females, and even these different relations is not consistent for all societies, in some countries height and RS is positively corrected for a particular gender but have negatively correlated in some other countries (8). The same study on TTP in South Africa showed that the proportion of planned pregnancies was 39% and the median TTP was 6 months (9). A cross-sectional study of time to first pregnancies was undertaken on Colombian women working in agricultural production (10). Fertility was measured using a discrete time analogue of Cox’s proportional hazard model, which showed that irregular relationships with partners, tobacco, and illness in the year prior to pregnancy and working in flower production are all associated with longer TTFP.

In human studies, there are often important biological variations among the unit experiments. This and not all of the biological variations can be accounted for covariates because some of them are unobservable. Ignoring unobservable variations may lead to an important bias for the effects of the observed covariates. Unobserved heterogeneity should be included in the statistical model to avoid biases. In this case, it can be reasonable to include a random effect part to account for unobserved biological factors and further heterogeneity. The traditional frailty and shared frailty models, described briefly in the next section, have so far described heterogeneity by random effect and by covariates. Frailty originates from gerontology where it is used to indicate that frail people have an increased risk of morbidity and mortality (11). 

In survival models, unobserved individual-level predictors may lead to biased estimation for model parameters, in order to refine these parameters, we can enter a random component) frailty( to the model and improve it (12). For continuous-time survival data with individual heterogeneity or clustered data, the mixed-effects survival models have been developed. These models are often termed frailty models, or survival models which include heterogeneity (13). Although more is known about the extent to which TTFP is associated with certain characteristics of women, there are limited analytical studies in rural districts in Iran. Despite its immense significance, no scientific community-based research on this event has so far been taken in Iran. To the best of our knowledge, no study in Iran has been done yet. Therefore, a frailty approach was used to study TTFP.

In this article, frailty models that incorporate both observable and unobservable factors are presented. Assuming that some unobservable latent variable causes that the residents of some rural have more chance to get pregnant earlier than the other area, made us to use frailty model and allow the baseline hazard to vary through different areas. 

## Materials and methods

In this cross-sectional study the target population was all couples planning and achieving conception in the rural areas of the Shiraz district (the south of Iran). It should be noted that the sterile part of the population was excluded from the study, and the first pregnancy of each woman and only planned pregnancies were included in this study. 

882 participants were selected using multi-stage cluster sampling between March 2014 and September 2015 from 18 health care centers. A self-administered questionnaire regarding the fertility history was used. The questionnaire was designed in such a way that the factors which applied to the pregnancy in general were asked. Menstrual age, maternal education level, adequate income (more than 1.5 million toman), women’s height, the age of marriage and regular menstruation before first pregnancy were assessed (irregular menstrual bleeding, absent menstrual bleeding, heavy menstrual bleeding, heavy and long-term menstrual bleeding and light menstrual bleeding were considered as irregular menstruation). 

**Ethical consideration**

This study was approved by the ethical committee of Shiraz University of Medical Sciences and the written consent form was obtained from all participants. 

**Statistical analysis**

Median or mean±SD was used for descriptive statistics. In this paper, the cumulative proportion of not conceiving at some points were assessed by the life-table approach. Comparisons of TTFP for different covariates were also based upon log-rank test. Kaplan-Meier curve was used to show the chance of pregnancy in two groups of regular and irregular menstruations. The term ‘pregnancy ratio’ was used for the hazard ratio regression coefficients. 

Frailty model: Cox regression is a usual model which is frequently used to analyze the time to event data, but this model can only explain the variability in observed time to the event. Misspecification or omitted factors can lead to further unexplained variability and the cox model cannot take account of these unexplained variabilities. 

There are two sources of variability and heterogeneities in such events. The first source is within-subject correlations which occur when some of individuals have more chance to experiencing the event (pregnancy) differently, and the second source is the event dependence correlation within each cluster (villages and families). A frailty model considers these sources of heterogeneities by modeling them as resulting from a latent by a multiplicative effect on the hazard baseline function. Hence, parametric frailty models were used to accommodate the rule of such clusters. Statistical analysis was done by SPSS 16 and Stata 10.0 software’s, p˂0.05 was considered statistically significant. 

## Results

**Figure 1:**
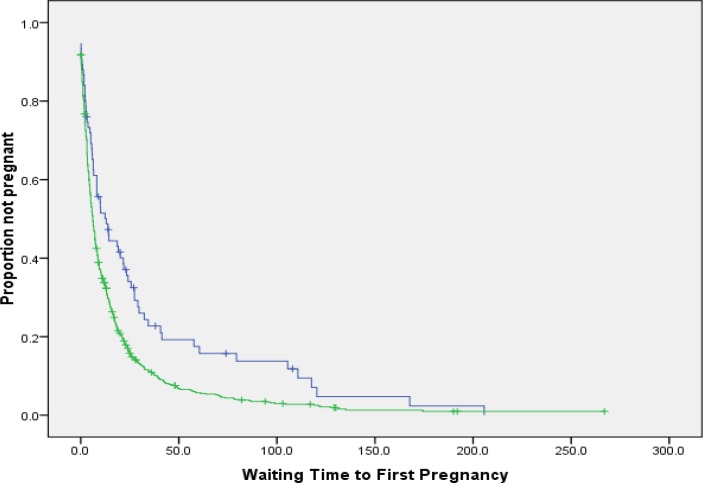
Kaplan–Meier plot of time to first pregnancy for irregular (upper curve) and regular (lower curve) menstruation

The mean age (SD) of the samples was 32.1±8.2 yr and married at the age of 18.4±4.0 with marriage duration of 13.8±9.2 (range 0-39.3). The median of TTFP was 6.4 months (inter-quartile range=15). The Live birth occurred in 93.0%, natural delivery in 87.2% and cesarean in 12% of mothers. Among the conceived women, the increasing age of marriage decreased TTFP (r=-0.126, p=0.001) but the increasing age at first conception increased the TTFP (r=0.332, p<0.001). The proportion of not conceiving at selected times are shown in table I. Infertility (inability to conceive after 1 yr of unprotected intercourse) rate was 35.5% and those with regular menstruation had more chance of conception (Figure 1). 

This study found no relationship between mother’s income and reproductive success in the rural population. There may be a tendency for small and tall women to have less reproductive success, but if there is such a tendency then it is very weak. The results of non-frailty and frailty models are shown in tables II, III, and IV. The maternal education, height, regularity of menstruation and age of marriage covariates seemed more related to TTFP, and were selected to be included in the models. 

The estimated θ in table II was significant (by likelihood ratio test, p<0.001) which indicated the presence of heterogeneity and necessitates the frailty models. The estimated hazard ratio for menstruation using the gamma frailty model was approximately 2. This is the estimated hazard ratio for two individuals having the same frailty in which one has regular menstruation and the other has irregular menstruation controlling for the other covariates in the model. In other words, a woman with regular menstruation is twice as likely to conceive at any time compared to a woman with irregular menstruation. In shared frailty model, the risk of getting pregnant at waiting time t for women with regular menstruation is about 70 percent more than women with irregular menstruation in the same cluster.

**Table I:** Life-table of duration of waiting time (months) to conception

**Variables**		**No.**	**Proportion of not conceiving at months**							**Median**	**p-value** **(Log-rank test)**
			**6**	**12**	**18**	**24**	**30**	**36**	**42**		
Age of marriage (yrs)											
	<15	152	0.65	0.48	0.38	0.30	0.26	0.21	0.16	11.2	0.006
	15-20	467	0.49	0.33	0.22	0.15	0.11	0.09	0.08	5.8	
	≥20	262	0.52	0.34	0.26	0.21	0.14	0.13	0.11	6.6	
	Total	881	0.52	0.36	0.25	0.19	0.15	0.12	0.10	6.8	
Menstruation											
	Irregular	75	0.65	0.51	0.44	0.35	0.26	0.23	0.19	13.2	0.001
	Regular	732	0.51	0.34	0.23	0.17	0.13	0.11	0.09	6.3	
Adequate Income											
	No	483	0.52	0.35	0.25	0.20	0.15	0.12	0.10	6.6	0.793
	Yes	396	0.53	0.36	0.25	0.18	0.14	0.12	0.11	7.0	
Maternal height (cm)											
	< 150	76	0.62	0.42	0.25	0.16	0.13	0.11	0.11	9.6	0.184
	150-160	445	0.51	0.34	0.24	0.17	0.13	0.11	0.09	6.5	
	160-170	315	0.51	0.34	0.27	0.20	0.15	0.14	0.12	6.3	
	≥ 170	37	0.55	0.48	0.34	0.34	0.27	0.23	0.19	10.5	

**Table II:** Results of time to first pregnancy (TTFP) by Weibull regression with no frailty, frailty and shared frailty models

** Maternal**	**No frailty**			**Frailty**			**Shared frailty**		
**Characteristics**	**HR**	**SE**	**p-value**	**HR**	**SE**	**p-value**	**HR**	**SE**	**p-value**
Education	0.97	0.036	0.449	0.94	0.056	0.312	0.97	0.041	0.529
Height (cm)	0.99	0.003	0.024	0.99	0.005	0.103	0.99	0.004	0.035
Age at menstruation (years)	0.98	0.023	0.279	0.98	0.035	0.663	0.98	0.024	0.554
Regular menstruation (yes-no)	1.68	0.228	<0.001	2.02	0.434	0.001	1.68	0.233	<0.001
Age at marriage ( years)	1.01	0.008	0.076	1.04	0.016	0.020	1.02	0.009	0.044
p	0.75	0.021		1.13	0.065		0.79	0.023	
θ	-	-		0.77	0.133		0.13	0.050	
Log-likelihood	-1282.75			-1247.08			-1264.55		
p (Likelihood ratio test for θ)	-			<0.001			<0.001		

HR=hazard ratio

SE= standard error

## Discussion

Although sexual health, contraception, and demographic reproduction have been widely studied in the south of Iran, this is the first study to describe fertility distribution as a representative of South-Iran population. Fertility was measured using TTFP. 

The validity and reliability of the questionnaire which used to collect the reproduction information was assessed in several studies and was suggested as suitable tool in retrospective studies (14, 15). In the present study, several factors were found to have a significant impact on TTFP. However, only the regularity of menstruation, age at marriage, and height of women had significant roles in the models. Furthermore, height and age at marriage played somewhat different roles in the last two models.

Although there are no data in Iranian population to compare these results with, the effect of age of women in this model is in agreement with some other studies. For example, in an observational study by Kaplan et al, 1,000 pregnant women were asked how long it took them to conceive (16). In a period of three months after marriage, about 71 percent of younger than 30 years’ old women were conceived. This proportion for older women was just 41%. Also, the result of an another study on 2,112 pregnant women in the UK showed that increasing age of both men and women have inverse correlation with the time to first pregnancy (17). 

The study by Amin and Bajracharya showed that a higher age at getting married had a negative correlation with TTFP, and when marriage took place during the peak fecundity years, women were more likely to be conceived sooner after marriage to compensate for their late start (18). Factors such as a lack of contraception, societal norms, and expectations supported short first-birth intervals. This view is supported by the findings of Singh *et al* (19). The interpretation of TTFP studies takes account of some possible behavioral factors. Our study shows that education levels do not have a significant association with TTFP. Contrary to our study, Joffe and Li showed that it is possible that the shorter TTFP observed among more educated couples was due to better public information being available on the timing of the fertile days in the women’s cycle (5). 

Also, Singh *et al* noted that there is a significant relation between the education levels of couples and the duration of the waiting time to conception (19). One explanation for this discrepancy could be the small sample sizes used in our study. There is, therefore, a need for further studies to explore this observation, with the aim of increasing planned pregnancies. The median TTFP was 6.4 months for our study. Since TTFP distribution has not been previously studied in Iran, there were no Iran-based studies to compare this distribution to. But it could be said that the study population had a relatively rapid rate of conception compared with some other studies. In research conducted in Manipur, India, the median duration of the waiting time to conception was eighteen months, which is a low rate compared to our study (19). 

In a South African study, the median time to pregnancy in the population was six months, with 68% of women achieving pregnancy in the first year, which is similar to our study (9). This proportion is within the 67-85% range reported for five European countries in a multi-country population study (20). An article on the trend for global infertility, published in 2009, noted that the rate of infertility ranges between 6-10% for some Western countries (21). Our data would suggest an association between the regularity of menstruation cycles and longer TTFP. This finding is in line with the previous findings of an early study in Bogota, Colombia which showed that irregular menstrual cycles can enhance TTP among women working in agricultural production (10). It is not possible to compare the figures with those for other population in Iran because there are no previous studies on this topic. Height had a significant effect on TTFP which is in agreement with a previous finding by Sear *et al* (8). 

This research was limited in several ways. A major critique concerning retrospective TTFP studies has been that they exclude women who have never been conceived. However, the strength of the present study is that we also included women who had tried to be conceived but up to that point had failed to do so. Design of the study may be lead to some biases which are inherent in retrospective studies, such as recall bias and measurement error, so a well-designed prospective study is suggested. Also we recommend to evaluate the effects of sexual behaviors and biological fecundity factors using a frailty time to event model. 

The disadvantage of parametric models is that they are not flexible in describing, for example, changes in the shape of the hazard function; for example, Weibull hazards are monotonous by definition. Parametric models can be made more flexible if the time axis is divided into intervals and allow the parameters of the model to be different on each interval (piecewise models) (11). The Pareto distribution for TTFP was used and discussed by Keding *et al* as satisfactory parametric model (22).

## Conclusion

This is the first comprehensive population based reproductive health study in the south of Iran. The median time to the first pregnancy in the population was 6.4 months. The TTFP result is probably influenced by the behavioral and demographic factors seen in other populations. Although information on several factors was available, the height, age of marriage and menstruation regularity seemed more important predictors of TTFP, which among them, age of marriage as a manageable factor is more highlighted. 

Distinguishing the factors influencing the dynamics of waiting time to first conception provides important information for the health planners, policy makers and researchers in reproductive health so that they can promote the status of elsewhere significant factors which are absent behind the targets to attain the national goal for fertility increase in the south of Iran and similar settings. The findings of this study also are applicable to informing intervention, sample size calculations, arousing new epidemiological research in reproductive health and planning for similar studies.
